# CD90 is not constitutively expressed in functional innate lymphoid cells

**DOI:** 10.3389/fimmu.2023.1113735

**Published:** 2023-04-11

**Authors:** Jan-Hendrik Schroeder, Gordon Beattie, Jonathan W. Lo, Tomasz Zabinski, Nick Powell, Joana F. Neves, Richard G. Jenner, Graham M. Lord

**Affiliations:** ^1^ School of Immunology and Microbial Sciences, King’s College London, London, United Kingdom; ^2^ Cancer Research UK (CRUK) City of London Centre Single Cell Genomics Facility, University College London Cancer Institute, University College London (UCL), London, United Kingdom; ^3^ Genomics Translational Technology Platform, University College London (UCL) Cancer Institute, University College London, London, United Kingdom; ^4^ Division of Digestive Diseases, Faculty of Medicine, Imperial College London, London, United Kingdom; ^5^ University College London (UCL) Cancer Institute, University College London, London, United Kingdom; ^6^ School of Biological Sciences, Faculty of Biology, Medicine and Health, Division of Infection, Immunity and Respiratory Medicine, University of Manchester, Manchester, United Kingdom

**Keywords:** innate lymphoid cell (ILC), CD90, intestine, DSS-colitis, fecal microbial transplant (FMT)

## Abstract

Huge progress has been made in understanding the biology of innate lymphoid cells (ILC) by adopting several well-known concepts in T cell biology. As such, flow cytometry gating strategies and markers, such as CD90, have been applied to indentify ILC. Here, we report that most non-NK intestinal ILC have a high expression of CD90 as expected, but surprisingly a sub-population of cells exhibit low or even no expression of this marker. CD90-negative and CD90-low CD127^+^ ILC were present amongst all ILC subsets in the gut. The frequency of CD90-negative and CD90-low CD127^+^ ILC was dependent on stimulatory cues *in vitro* and enhanced by dysbiosis *in vivo*. CD90-negative and CD90-low CD127^+^ ILC were a potential source of IL-13, IFNγ and IL-17A at steady state and upon dysbiosis- and dextran sulphate sodium-elicited colitis. Hence, this study reveals that, contrary to expectations, CD90 is not constitutively expressed by functional ILC in the gut.

## Introduction

Resident leukocytes play an important role in maintaining mucosal surfaces at steady state and early during an infection (1, 2). Since the discovery of innate lymphoid cells (ILC) about a decade ago, it has become increasingly apparent that these cells play a significant role in mucosal homeostasis. However, the role for ILC is far from being fully characterized, and much of the current knowledge has been gained from testing concepts that had previously been established for T and NK cell biology. As such, group 1, 2 and 3 ILC (ILC1, ILC2 and ILC3) express T-bet, GATA3 and RORγt, respectively, as characteristic transcription factors as well as cytokines associated with Th1, Th2 and Th17 cells (1, 3). Due to the absence of T cell receptor (TCR) expression in ILC, these cells elicit immune functions in response to cytokines, chemokines and neurotransmitters, as has been well described for NK cells (1, 2).

Similarly to T and NK cells, ILC express the glycophosphatidylinositol (GPI) anchored protein CD90 in diverse tissues, and CD90 has often been used as a key marker to identify ILC (4–24) or as key target to deplete ILC in Rag-deficient mice using a specific antibody (e.g. 25–32). Despite the presence of CD90 on T and NK cells, very little is known regarding its functionality (5). In NK cells, CD90 downregulation was associated with successful differentiation, but its presence has also been linked to an activation phenotype (33–35). IL-17A-producing inflammatory ILC2 in lungs and small intestinal lamina propria (SI LP) have been observed to have lower expression of CD90 in comparison to natural ILC2, but the implications of this are not known (36–38). In relation to this, transition of CD90^low^ to CD90^high^ ILC2 precursors has been described using an *in vitro* model in which CLP were seeded, but again the role of the gain in CD90 is unknown (39). Furthermore, IL-10 expressing intestinal ILC2 have a characteristic lack of CD90 expression (40). ILC3 from the intestinal lamina propria of naïve mice were reported to have a characteristic CD90^high^ CD45^low^ phenotype, however, ILC3 were also found among CD90^low^ CD45^high^ ILC from the small intestine (41). Recently, it was reported that in the murine liver Ly49E^+^ ILC1 have a lower expression of CD90 than Ly49E^-^ ILC1 (42, 43).

Here, we report for the first time that cytokine-producing intestinal lamina propria ILC exhibit varied expression of CD90, and strikingly some ILC show no expression of this marker. These CD90^-^ and CD90^low^ ILC are a significant source of IFNγ, IL-13 and IL-17A upon dysbiosis and dextran sulphate sodium (DSS)-elicited colitis. However, in naïve mice, CD90^-^ ILC have a dominant type 2 cytokine expression profile. Furthermore, stimulation with IL-25/IL-33 promotes the frequency of CD90^-/low^ ILC2 *in vitro*. Conversely, IL-12/IL-18 stimulation results in a lower prevalence of CD90^-/low^ NKp46^+^ ILC. These data suggest that CD90 expression in intestinal ILC is regulated by cytokines and has a limited suitability as a constitutive marker of the ILC lineage.

## Results

### CD90-negative colonic lamina propria CD127^+^ ILC produce cytokines upon induced colitis

**Figure 1:**
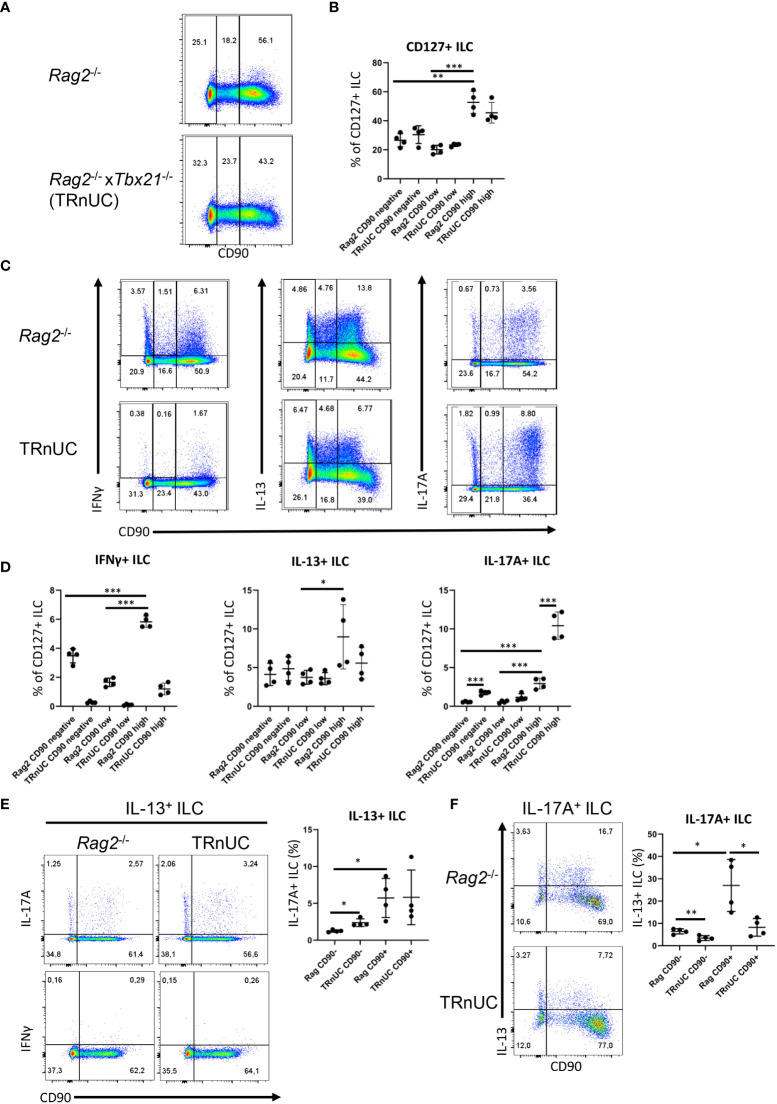
CD90-negative Rag2-deficient ILC are a substantial source of IFNγ and IL-13 during DSS colitis. cLP ILC from 5% DSS-treated *Rag2* ^-/-^ and TRnUC mice were isolated and stimulated with PMA and ionomycin (3 hours) prior to flow cytometry analysis. **(A)** Frequencies of CD90^hi^, CD90^low^ and CD90^-^ in total CD127^+^ ILC and **(B)** statistical analyses are shown. **(C)** IFNγ, IL-13 and IL-17A expression in CD90^hi^, CD90^low^ and CD90^-^ total CD127^+^ ILC and **(D)** corresponding statistical analyses are outlined. **(E)** CD90 co-expression with IL-17A or IFNγ in IL-13^+^ ILC and corresponding statistical analyses are shown. **(F)** Flow cytometry and statistical analysis of CD90 and IL-13 expression in IL-17A^+^ ILC are presented. Data shown are representative of 4 biological replicates. *p < 0.05; **p< 0.01; ***p<0.001.

CD90 expression in ILC was tested in a mouse model of DSS-induced colitis. BALB/c *Rag2* ^-/-^ mice were treated with 5% DSS in the drinking water for 5 days after which the animals showed clinical signs of colitis like weight loss (44), and the cytokine expression profile of colonic lamina propria (cLP) ILC was analyzed at day 10. Analyses of CD45^+^ Lin^-^ (CD3, CD5, B220, CD19, CD11b, TER-119, Gr-1, FcϵRI) CD127^+^ cLP ILC re-stimulated with PMA and ionomycin (PMA Iono) *in vitro* revealed that in addition to CD90^high^ ILC there were CD90^-^ and CD90^low^ ILC populations (**Figure 1A**; **Supplementary Figures 1A, B**).

The abundance of CD90^high^ ILC was greater than that of CD90^-^ and CD90^low^ ILC, but these populations represented ~30% and 20%, respectively, of the total CD127^+^ ILC population. In order to determine whether CD90^-^ and CD90^low^ ILC were associated with a T-bet-expressing ILC subset, we analyzed CD90^-^ and CD90^low^ ILC in *Tbx21* ^-/-^ x *Rag2* ^-/-^ non-ulcerative colitis (TRnUC) mice. This revealed that the presence of these cells was not dependent on T-bet, and their frequency was not affected. CD90^-^ and CD90^low^ ILC were a relevant source of IFNγ, IL-13 and IL-17A, but still significantly less potent than CD90^high^ ILC in these DSS-treated *Rag2*-deficient mice (**Figures 1C, D**). DSS-treated TRnUC mice did not have altered frequencies of CD90^-^ and CD90^low^ ILC or IL-13 production in these cells in comparison to DSS-treated *Rag2* ^-/-^ mice (**Figures 1B–D**). However, TRnUC mice had a greater frequency of IL-17A expressing CD90^-^ and CD90^high^ ILC than *Rag2* ^-/-^ mice. This could be explained by the far greater cellularity of ILC3 in *Rag2* ^-/-^ mice driven by the deficiency of *Tbx21* (45).

ILC2 expressing RORγt were reported to have no or lower expression of CD90 in comparison to RORγt-negative natural ILC2 (36, 37). We detected CD90^-^ and CD90^+^ ILC co-expressing IL-13 and IL-17A (**Figures 1E, F**). We also detected more CD90^+^ than CD90^-^ inflammatory IL-13^+^ IL-17A^+^ ILC2 (**Figures 1E, F**), supporting the notion that inflammatory ILC2 have a CD90^-^ and CD90^+^ phenotype. We also noted that CD90^-^ ILC can express IL-17A independently of IL-13 (**Figure 1F**). Interestingly, T-bet-deficiency appears to promote the frequency of CD90^-^ IL-17^+^ among IL-13^+^ ILC2 in these *Rag2* ^-/-^ mice.

Functional CD90^-^ and CD90^low^ ILC were also observed in DSS-treated wild-type BALB/c mice (**Supplementary Figures 2A, B**). In these DSS-treated mice, CD90^high^ ILC were a vastly more significant source of IFNγ, IL-13 and IL-17A in comparison to CD90^-^ and CD90^low^ ILC (**Supplementary Figures 2C, D**). As observed in Rag2-deficient mice, CD90^-^ and CD90^low^ ILC were able to produce IL-17A and IL-13, but the proportion of CD90^-^ and CD90^low^ ILC producing these cytokines was increased in DSS-treated BALB/c-background *Tbx21* ^-/-^ mice (**Supplementary Figures 2C, D**). These *Tbx21* ^-/-^ mice also had an enhanced frequency of IL-17A^+^ CD90^high^ ILC (**Supplementary Figures 2C, D**). CD90^-^ and CD90^low^ ILC were also detected in DSS-treated WT C57BL/6 mice (**Supplementary Figures 3A, B**). As observed in the other mouse strains, CD90^-^ and CD90^low^ ILC produced IFNγ, IL-13 and IL-17A, although CD90^high^ ILC appeared to be a greater source of these cytokines (**Supplementary Figures 3C, D**). In contrast to BALB/c background mice, C57BL/6 background *Tbx21* ^-/-^ mice did not have a greater prevalence of IL-17A- and IL-13-producing CD90^-^, CD90^low^ or CD90^high^ ILC than WT mice, however, the frequency of CD90^low^ ILC was reduced significantly (**Supplementary Figures 3A–D**).

Furthermore, we did not detect any IFNγ producing IL-13^+^ ILC in contrast to IL-17A production among CD90^+^ and CD90^-^ IL-13^+^ ILC2 in DSS-treated BALB/c *Rag2* ^-/-^ mice and C57BL/6 WT mice (**Figures 1E, F**; **Supplementary Figure 3E**). These data indicate that low expression of CD90 is not a simple marker of inflammatory ILC2 in these mice.

### CD90-negative CD127^+^ ILC have a predominant type 2 phenotype at steady state

Similar to DSS-treated mice (**Figure 1**; **Supplementary Figures 2**, **3**), most ILC were CD90^high^ in naïve untreated C57BL6 mice. However, CD90^-^ and CD90^low^ ILC populations were also detected in these mice (**Supplementary Figures 4A, B**). Interestingly, both CD90^-^ and CD90^low^ ILC produced predominately IL-13 and IL-5 and fewer of these cells produced IFNγ and IL-17A (**Supplementary Figures 4B–G**). Although moderately low, CD90^-^ and CD90^low^ ILC had a significantly greater frequency of IFNγ positivity than CD90^high^ ILC (**Supplementary Figure 4C**). A similar trend was not observed for IL-17A (**Supplementary Figure 4D**). IFNγ and IL-17A production was also driven mostly by distinct populations of cells (**Supplementary Figure 4B**). Further analyses revealed that the prevalence of IL-13^+^ and IL-5^+^ ILC was greater among CD90^high^ and CD90^low^ ILC in comparison to CD90^-^ ILC (**Supplementary Figures 4B–G**, **5A**). *Tbx21* ^-/-^ CD90^-^, CD90^low^ and CD90^high^ ILC exhibit greater expression of IL-5 than ILC in WT mice (**Supplementary Figures 4B–G**), which could be explained by one of our previous reports indicating increased cLP ILC2 abundance in *Tbx21* ^-/-^ mice (46). Since CD90^-^ and CD90^low^ ILC appeared to be predominately functional ILC2, we sought to determine whether these cells were able to adopt functional characteristics of ILC1 and ILC3. Plasticity of ILC2 allowing expression of T-bet and RORγt is a well-known phenomenon (1). Similar to the observations in DSS-treated WT C57BL/6 and *Rag2*-deficient mice, we detected minimal co-expression of IL-13 and IL-17A in CD90^-^ and CD90^low^ cLP ILC from naïve WT and *Tbx21* ^-/-^ mice indicating the presence of a minor inflammatory ILC2 population (**Supplementary Figure 5A**). However, we could also find IL-13 and IL-17A co-expressing CD90^high^ ILC. In contrast, virtually no IL-13 and IFNγ co-expressing CD90^-^ and CD90^low^ ILC were detected in these mice (**Supplementary Figure 5B**).

### CD90 expression in CD127^+^ ILC is controlled by stimulatory cues

**Figure 2:**
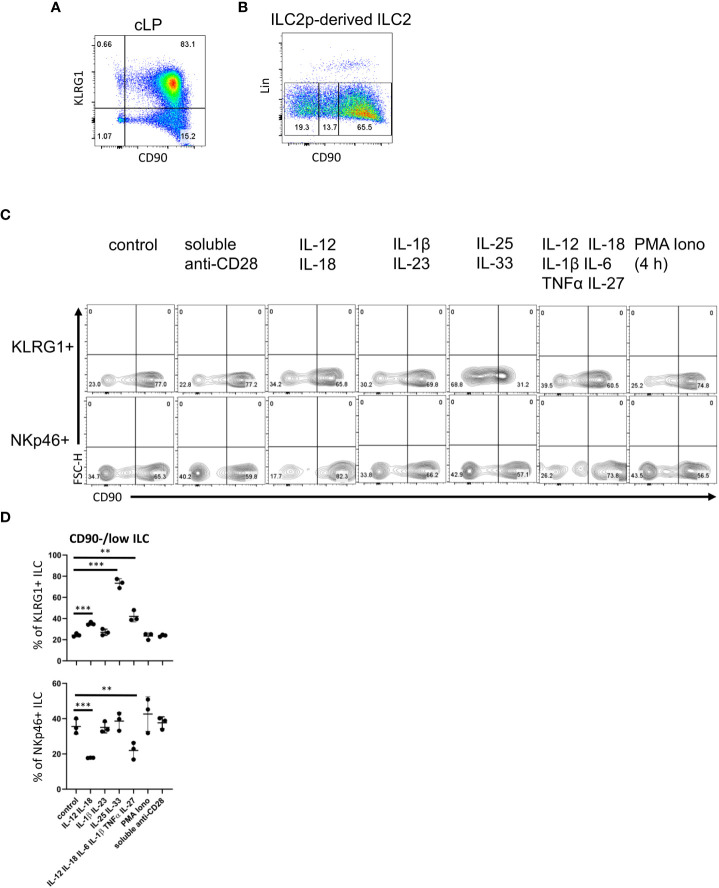
cLP ILC have a variable expression of CD90 depending on stimulatory cues. **(A)** KLRG1 and CD90 co-expression in cLP CD127^+^ ILC was demonstrated by flow cytometry (n=12). **(B)** ILC2 were generated from ILC2p stimulated with IL-7, SCF and IL-33, and seeded onto OP9-DL1. CD90^hi^, CD90^low^ and CD90^-^ ILC2 are shown. **(C, D)** KLRG1^+^ or KLRG1^-^ CD127^+^ ILC were isolated and stimulated *in vitro* for 48 hours prior to harvest and flow cytometry analyses of KLRG1^+^ or NKp46^+^ ILC, respectively. In addition to a control condition, soluble agonistic anti-CD28 antibodies, IL-12&IL-18, IL-1β&IL23, IL-25&IL-33 or IL-12&IL-18& IL-1β&IL-6&TNFα &IL-27 were used as stimuli. In a separate condition designated as “PMA Iono”, sorted cells were stimulated with PMA and ionomycin in the presence of monensin for the final 4 hours prior to harvesting. **(D)** Flow cytometry analyses of CD90^hi^ and CD90^low/neg^ CD127^+^ ILC and statistical analyses of CD90^low/neg^ ILC frequencies among KLRG1^+^ or NKp46^+^ cLP ILC are outlined. Data shown are representative of 3 biological replicates. **p< 0.01; ***p<0.001.

Overall, we detected CD90^-^ and CD90^low^ ILC in both untreated and DSS-treated mice. This suggests that CD90 is not a reliable marker for detection of all ILC in the gut. When we analyzed CD127 and CD90 co-expression in lineage-negative cLP leukocytes, we noticed that almost all CD90^+^ cLP ILC had a detectable surface expression of CD127 in naïve C57BL/6 WT and DSS-treated C57BL/6 WT, BALB/c WT and BALB/c *Rag2* ^-/-^ mice (**Supplementary Figure 6**). For further analyses, KLRG1 was used as a marker of intestinal ILC2 in line with recent publications (37, 45–47). The use of KLRG1 as a marker for intestinal ILC2 has an advantage over GATA3 as intestinal ILC3 have a low expression of GATA3 and the expression of this transcription factor is variable within the ILC2 population (48–50). KLRG1^hi^ intestinal ILC as gated in this study require GATA3 for post-developmental maintenance, supporting the notion these cells are ILC2 (51). We found that CD90^-^ and CD90^low^ ILC can be detected among both KLRG1^hi^ and KLRG1^-^ cLP ILC from C57BL/6 background mice, demonstrating that CD90^-^ and CD90^low^ ILC are also components of the non-ILC2 compartment (**Figure 2A**).

Next, following an established method to develop ILC2 *in vitro*, we seeded bone marrow-derived ILC2 precursors (ILC2p; defined as Lin^-^ CD127^+^ α4β7^hi^ Flt3^-^ CD25^+^) in a 6-day culture on OP9-DL1 stromal cells in the presence of IL-7, SCF and IL-33 (52). Strikingly, the Lin^-^ cell population that was generated also exhibited variable levels of CD90 (**Figure 2B**). Most of the ILC were CD90^high^, but there were also substantial CD90^-^ and CD90^low^ subpopulations.

In order to determine whether CD90 expression can be altered by immunological stimulations, we isolated KLRG1^+^ cLP ILC2 and KLRG1^-^ cLP ILC for *in vitro* culture with OP9-DL1 cells in the presence of distinct cytokines. Strikingly, ILC2 stimulation with IL-25 and IL-33 induced the presence of CD90^-/low^ ILC2 (**Figures 2C, D**; **Supplementary Figure 7**). A similar but less potent effect was observed when IL-12 and IL-18 were added to the culture medium. Additional IL-6/IL-1β/TNFα/IL-27 stimulation did not further alter IL-12/IL-18-mediated CD90^-/low^ ILC2 frequency, while IL-1β/IL-23 stimulation also had no effect. Conversely to ILC2, IL-12/IL-18 stimulation of non-ILC2 in the presence or absence of IL-6/IL-1β/TNFα/IL-27 resulted in fewer CD90^-/low^ NKp46^+^ ILC (**Figures 2C, D**). IL-1β/IL-23 and IL-25/IL-33 stimulation of these cells had no effect in terms of CD90 expression. Stimulation with PMA and ionomycin or a soluble agonistic anti-CD28 antibody [chosen due to reports of its expression in human ILC (53, 54)] also had no effect on the frequency of CD90 expressing ILC2 or NKp46^+^ non-ILC2.

### All ILC subset populations in the intestine exhibit variable levels of CD90

**Figure 3:**
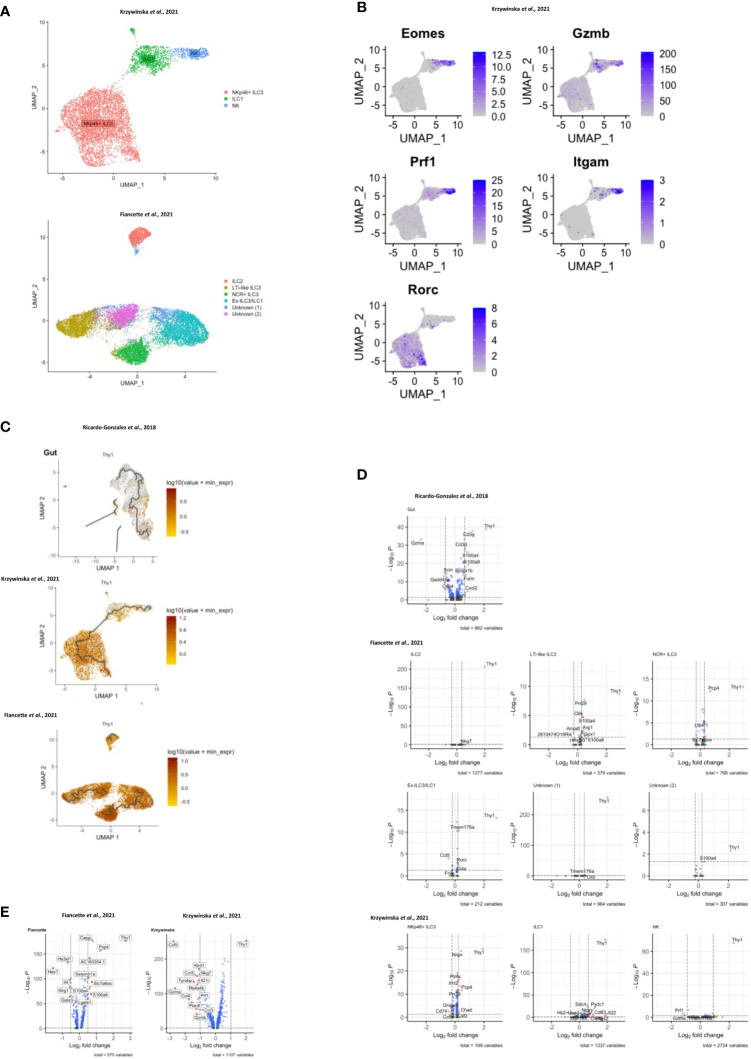
Transcriptomic analyses of CD90 expression in intestinal ILC. scRNA-seq data sets of intestinal ILC from published studies (47, 49, 55) were employed to analyze expression of *Thy1* (encoding CD90) across ILC subsets and its role on the global transcriptional profile. **(A)** UMAP plots of ILC subset annotation from the scRNA-seq data sets of the (47, 55) studies. **(B)** UMAP analyses of gene expression in the ILC subset clusters in the data set obtained from (55). **(C)** UMAP analysis of *Thy1* expression intensity in ILC subsets in the respective studies. A trajectory analysis along the *Thy1* expression intensity was performed in the indicated ILC subsets. **(D, E)** Volcano plots comparing gene expression (log2 fold change and p_adj_) between *Thy1* ^high^ ILC versus *Thy1* ^low/negative^ **(D)** ILC subsets, as annotated in the respective published data set, and **(E)** total ILC. The most differentially expressed genes are labelled. In order to generate the volcano plots the median normalized *Thy1* expression across all datasets was calculated and used to delineate *Thy1* ^high^ and *Thy1* ^low/negative^ cells.

In order to investigate CD90 variation in ILC more closely, we analyzed single-cell (sc)RNA-seq data sets from three recent publications: ILC2 from gut, skin, lung, fat and bone marrow (BM) (Ricardo-Gonzalez et al. (49)), intestinal ILC2, LTi-like ILC3, NKp46 (NCR)^+^ ILC3 and ex-ILC3/ILC1 (47), and intestinal NK cells, ILC1 and NKp46^+^ ILC3 (55) (**Figures 3A–C**; **Supplementary Figure 8A**). Visualising clusters of cells that have similar transcriptional profiles using uniform manifold approximation and projection (UMAP) dimensionality reduction and overlaying expression levels of *Thy1* (encoding CD90), we found that *Thy1* expression could be detected across all of the ILC subsets in each dataset (**Figure 3C**; **Supplementary Figure 8A**). A pseudotime trajectory analysis of these ILC subsets did not uncover a specific developmental direction from either *Thy1* high to low expression or vice versa (**Figure 3C**; **Supplementary Figure 8A**). Identification of genes up and downregulated in cells positive for CD90 mRNA vs negative/low for CD90 mRNA within each dataset only identified a limited set of genes (**Figure 3D**; **Supplementary Figure 8B**). Together with the expression of CD90 across the various cell clusters, this indicates that CD90^-/low^ ILC are not a novel ILC population with their own expression profile. In terms of ILC2, the Fiancette et al. data set indicated a higher expression of *Nkg7* in CD90 mRNA-high cells, but no genes specific for CD90 mRNA-negative/low ILC2 were detected in this data set. In contrast, in the Ricardo Gonzalez et al. data set intestinal CD90 mRNA-negative/low ILC2 exhibited greater expression of *Gzma* (encoding granzyme A) and *Gdd45a*, *Scin* and *Ctla4*, while intestinal CD90 mRNA-high ILC2 were characterized by *S100a4*, *S100a6*, *Cd3d*, *Cd3g*, *Furin* and *Cxcl2* expression. *S100a4* and *S100a6* expression was also detected in CD90 mRNA-high ILC2 from fat, while *S100a4* and *S100a6* was exhibited in cutaneous and pulmonary CD90 mRNA-high ILC2, respectively. *Lgals1* expression was detected in CD90 mRNA-high ILC2 from lungs, skin and fat tissue. As observed in the Fiancette et al. data on intestinal ILC2, *Nkg7* expression is also associated with CD90 mRNA-high ILC2 from skin and bone marrow, in addition to *Cd7*, *Ncr1*, *Klrk1*, *Ms4a4b* and *Ccl5* in BM CD90 mRNA-high ILC2. No genes showed consistently higher expression in CD90 mRNA-negative/low cells across all the tissue types but, in the bone marrow, CD90 mRNA-negative/low ILC2 were associated with the expression of *Hbb-bs*, *Hbb-b7*, *Hba-a1*, *Hba-a2* and *S100a8*. The Fiancette et al. data set revealed a characteristic expression of *S100a4*, *S100a6*, *Pm29* and *Arg1* in CD90 mRNA-high LTi-like ILC3, while genes specific for CD90 mRNA-negative/low LTi-like ILC3 were not detected. Both the Fiancette et al. and Krzywinska et al. data sets highlight a specific expression of *Pcp4* in CD90 mRNA-high NKp46^+^ ILC3, while the latter data set also indicate an expression of *Nrgn* in CD90 mRNA-high NKp46^+^ ILC3 and *Cd74* in CD90^-/low^ NKp46^+^ ILC3. In terms of the ex-ILC3/ILC1 cluster *Tmem176a*, *Rorc* and *Gda* expression was enhanced in CD90 mRNA-high cells, while *Ccl5* expression was more common in cells in which CD90 mRNA was absent or low. In the Krzywinska et al. data, CD90 mRNA-high ILC1 exhibited a characteristic expression of *Il22*, *Cd83* and *Pxdc1*, while CD90 mRNA-negative/low ILC1 were not defined by specific genes. No genes were found to be upregulated in CD90 mRNA-high NK cells but *Prf1* and *Gzma* expression was enhanced in CD90 mRNA-negative/low NK cells. Further analyses demonstrated that also only a very few genes were specific for CD90 mRNA-negative/low and CD90 mRNA-high in total ILC and NKp46^+^ ILC (**Figure 3E**). As similar sets of genes were associated between CD90 mRNA-negative/low and CD90 mRNA-high ILC subsets, it appears that these respective populations may be related.

### Dysbiosis correlates ILC1 and ILC3 lymphopenia and altered CD90 expression in ILC

**Figure 4:**
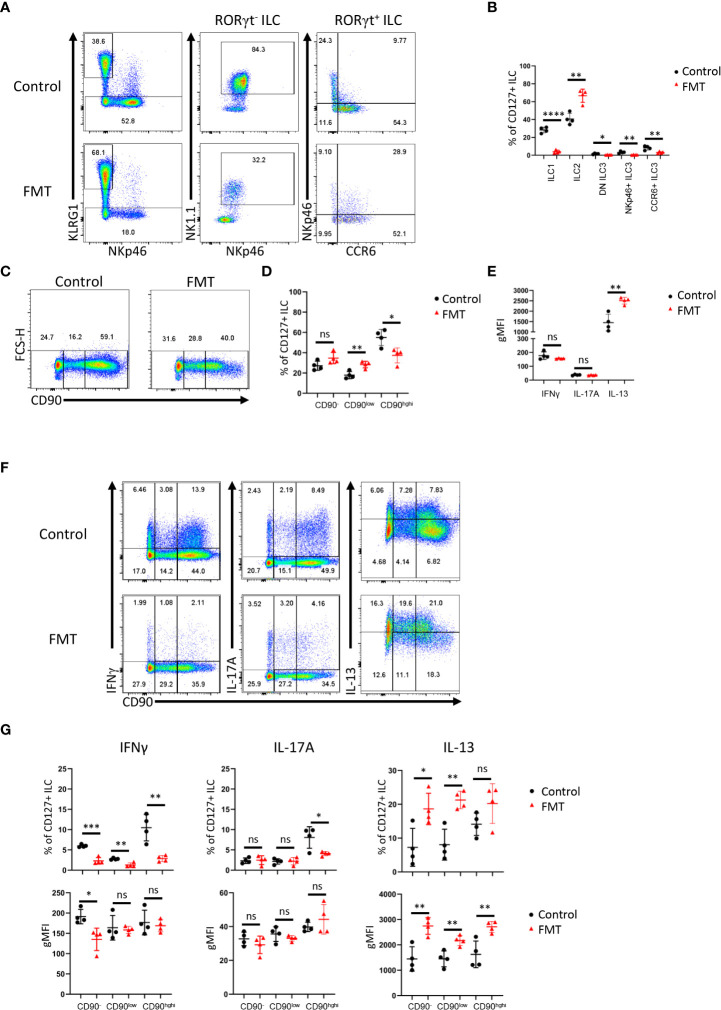
Dysbiosis-triggered appearance of functional cLP ILC with a low expression of CD90. Feces from TRUC mice were transferred into *Rag2* ^-/-^ mice and cLP leukocytes were isolated 21 days later from treated and untreated mice. **(A)** KLRG1^+^ ILC2, KLRG1^-^ RORγt^-^ NKp46^+^ NK1.1^+^ ILC1, KLRG1^-^ RORγt^+^ ILC3 subsets from FMT-treated and untreated control mice were analyzed by flow cytometry. ILC3 subsets were defined as NKp46^+^ CCR6^-^, CCR6^+^ NKp46^-^ or DN (‘double negative’) in these analyses. **(B)** A statistical analysis of ILC subset frequency among the whole cLP ILC population is outlined. **(C)** ILC with no or a low or high expression of CD90 were analyzed by flow cytometry and **(D)** a statistical analysis of the frequency of these ILC among the whole ILC population is presented. **(E)** The per cell expression of IFNγ, IL-17A and IL-13 in ILC was analyzed statistically. **(F)** IFNγ, IL-17A and IL-13 expression in CD90^-^, CD90^low^ and CD90^high^ ILC was determined by flow cytometry. **(G)** Related statistical analyses investigating the frequency of respective ILC and the per cell expression of IFNγ, IL-17A and IL-13 in the CD90^-^, CD90^low^ and CD90^high^ ILC populations are shown. Data are representative of 4 biological replicates. ns, non-significant; *p < 0.05; **p< 0.01; ***p<0.001; ****p<0.0001.

Next, we sought to further analyze CD90 expression dynamics in a model of dysbiosis-driven spontaneous colitis in *Rag2* ^-/-^ mice. We have previously shown that spontaneous colitis in *Tbx21* ^-/-^ x *Rag2* ^-/-^ ulcerative colitis (TRUC) mice is partially driven by IL-17A-producing CD90^+^ ILC (25, 56). Hence, it was anticipated that these ILC would also promote inflammation in *Rag2* ^-/-^ mice receiving a transfer of feces derived from TRUC mice. These mice developed colitis with decreased body weight and increased colon weight (data not shown). However, in contrast, we detected reduced frequency of DN ILC3, CCR6^+^ ILC3, NKp46^+^ ILC3 and ILC1 (**Figures 4A, B**; **Supplementary Figure 1A**). Hence, ILC2 formed a large proportion of the cLP ILC upon fecal microbial transfer (FMT). In addition to these ILC subset frequency alterations, we detected fewer CD90^high^ and more CD90^low^ cells among the ILC population upon FMT treatment, but the frequency of CD90^-^ ILC was not altered in these mice (**Figures 4C, D**). Consistent with a greater frequency of ILC2 in FMT-treated mice, cLP ILC production of IL-13 was enhanced, while a significant alteration in IFNγ or IL-17A production was not detected (**Figure 4E**). However, the frequency of IFNγ producing CD90^high^, CD90^low^ and CD90^-^ ILC was much reduced upon the enforced dysbiosis (**Figures 4F, G**). Furthermore, pathogenic FMT also resulted in a lower frequency of IL-17A^+^ CD90^high^ ILC, while IL-17A production in CD90^low^ and CD90^-^ ILC was not affected. When comparing CD90^high^, CD90^low^ and CD90^-^ ILC that produced IFNγ and IL-17A, only a reduction in IFNγ production in CD90^-^ ILC was observed. In contrast to IFNγ and IL-17A, FMT appeared to promote IL-13 production in CD90^high^, CD90^low^ and CD90^-^ ILC subsets.

## Discussion

Ever since the discovery of ILC around a decade ago, there have been refinements to the ILC analysis strategy by flow cytometry. This is still an active process, as an increasing number of functional states within the ILC subsets are being reported. In the past, many groups have used CD90 as a marker for ILC and CD90-specific antibodies are often employed to deplete ILC *in vivo* (e.g. 25, 27–32). However, our results demonstrate that the use of CD90 to detect and purify ILC has limitations when analyzing intestinal populations. In contrast to the notion that CD90 is a pan-ILC marker, the data presented in this study reveal that intestinal ILC can be separated into CD90^-^ and CD90^high^ ILC in addition to CD90^low^ ILC, which are most likely transitional cells. CD90^-^ ILC2 were also detected in the lungs indicating that the findings in our study are applicable to ILC from diverse tissues (57). In our hands, CD127 is a far more reliable marker of ILC than CD90. Virtually all CD90^+^ cLP ILC express CD127, however other reports indicate that pulmonary ILC can lose CD127 *in vivo* and IL-7 downregulates CD127 expression in ILC *in vitro* (27, 58). Hence, in the absence of better ILC markers, we advise using a combination of CD127 and CD90 to detect ILC.

In BALB/c background mice, CD90^-^ ILC accounted for about a fifth to a third of cLP ILC, and we detected a substantial amount of IFNγ, IL-13 and IL-17A production by these cells in the context of DSS- or dysbiosis-elicited colitis. Hence, we believe these findings support the notion that these cells play a relevant role in the ILC response in intestinal tissue. It is out of the scope of this report to define a functionality of CD90 in ILCs, but it was striking to note that whilst ILC2 accounted for most cytokine-producing cLP CD90^-^ ILC in C57BL/6 at steady state, the lack of CD90 expression was not restricted to ILC2. The combination of IL-33 and IL-25, known to activate ILC2, was a potent stimulus for CD90 downregulation in cLP ILC2 *in vitro*, suggesting that low CD90 expression may be an indicator of intestinal ILC2 activity. In this experiment CD90 in sorted ILC2 was reduced within a relatively short culture period of 48 hours indicating that CD90 expression is dynamic. Interestingly, CD90 expression in pulmonary ILC2 was also shown to drop upon stimulation with IL-33 (59). Furthermore, distinct ILC2 clusters from adult and neonate lungs with high and low expression of CD90 were detected by scRNAseq analysis (60). This publication also presents a trajectory analysis predicting transformation of adult pulmonary ILC2 along these clusters which may also indicate a dynamic expression of CD90 in ILC2. In our report cLP ILC2 stimulation with IL-12 and IL-18 also enhanced the frequency of CD90^-/low^ cells. It has been reported that IL-12/IL-18 and IL-25/IL-33 can induce ILC2 to express T-bet and RORγt, respectively (37, 61). In a model of DSS-induced colitis, we could not associate either IFNγ or IL-17A production by cLP ILC2 with loss of CD90 expression. In contrast to ILC2, CD90 expression was enhanced by IL-12/IL-18-mediated stimulation in NKp46^+^ cLP ILC, which may further indicate that CD90 plays a functional role. Furthermore, in dysbiotic mice we noticed a reduced expression of CD90 in IL-13-producing ILC indicating that CD90 downregulation occurs in activated ILC2 in these mice. Such modified expression of CD90 upon exposure to pathogens is not without precedent. The frequency of intestinal CD90^-^ ILC2 was enhanced in *Hoil1* ^-/-^ mice, a mouse model defined by microbe-driven intestinal inflammation (62). In comparison, an alteration of CD90^+^ ILC2 prevalence was not observed in these mice (62). Furthermore, *Aspergillus fumigatus*-induced inflammation also leads to the promoted occurrence of pulmonary CD4^+^ T cells with low expression of CD90 (63). In the intestine, variable expression of CD90 can be observed in Vγ7^+^ intraepithelial lymphocytes in addition to conventional CD4^+^ and CD8^+^ T cells (64, 65).

The functional role of CD90 expression on murine ILC is unknown and is also ill-defined in other lymphocytes, while CD90 expression in human ILC appears to be lacking. Known ligands of CD90 are integrins αvβ3, αxβ2, α_M_β2, α_5_β1, α_V_β5, syndecan-4 and CD97, and interactions with binding partners have reported to occur either *in cis* or *in trans* (4, 66–69). *In vitro* studies in unpolarized and polarized CD4^+^ T cells suggested that CD90 activation with a specific antibody can promote proliferation as well as IFNγ, IL-17A and IL-13 production, in particular in the case of co-stimulation with an agonistic anti-CD28 antibody in the absence of TCR stimulation (70, 71). Further work is required to determine the significance of this signaling axis, but, strikingly, scRNA-seq analysis in germinal center (GC) T follicular helper (T_FH_) cells showed distinct transcriptional differences between cells with high expression of CD90 versus cells with low or no expression of CD90 (72). These differences included high expression of genes indicative of exocytosis/degranulation in CD90^-/low^ GC T_FH_ cells, and genes relating to chemokine receptors and proliferation in CD90^high^ GC T_FH_ (72). Moreover, in addition to CD90^high^ CD8^+^ T cells, splenic CD90^-^ and CD90^low^ CD8^+^ T cells are also a relevant source of IFNγ in a mouse model of LCMV infection (73). The CD90 extracellular domain has binding sites for αvβ3 and syndecan-4, which may be the basis of a reported *in trans* interaction of CD90 with αvβ3 and syndecan-4 expressed on other cells (4, 74). Indeed, the interaction between CD90 and αvβ3 was functional in CD4 T cells in terms of promoting the differentiation of Th2 cells (74). Binding sites for the *in trans* interaction with other integrins or CD97 are yet to be characterized. In addition to *in trans* interactions, αvβ5 is inactivated by binding CD90 *in cis*, preventing activation of latent TGF-β1 (4, 75). Cis CD90-CD90 interactions have been suggested to promote cluster formation in lipid rafts, which may play a critical role for RhoA-dependent signaling, as reported downstream of CD90 (4, 69). Due to its numerous known ligands, CD90 may equip ILC for intercellular interactions with several hematopoietic or non-hematopoietic cell types, but the functional role of CD90 for ILC has still to be defined (5). Interestingly, CD90 was demonstrated to regulate PPARγ expression in adipocytes (76), and other groups have reported previously that PPARγ plays an important role in ILC2 functionality (77, 78). Our study marks the first step to defining CD90 function in ILC by revealing that intestinal ILC can be separated into CD90^+^ and CD90^-^ populations. These data have critical implications for the analysis procedures through which ILC functionality will be uncovered in intestinal tissue.

## Methods

### Animals

C57BL/6 WT, *Tbx21* ^-/-^ (both C57BL/6 and BALB/c background) and *Rag2* ^-/-^ (BALB/c background) mice were sourced commercially (Charles River). A colony of colitis-free BALB/c *Rag2* ^-/-^ x *Tbx21* ^-/-^ (TRnUC) mice was generated from a descendant of the TRUC colony described previously (25, 56, 79). All mice were housed in specific pathogen–free facilities at King’s College London Biological Services Unit or at Charles River Laboratories.

### Isolation of cells

cLP leukocytes were isolated using a published method (80). Briefly, the epithelium was removed by incubation in HBSS lacking Mg^2+^ or Ca^2+^ (Invitrogen) supplemented with EDTA and HEPES. The tissue was further digested in 2% of fetal calf serum (FCS Gold, PAA Laboratories) supplemented in 0.5 mg/ml collagenase D, 10 μg/ml DNase I and 1.5 mg/ml dispase II (all Roche). The LP lymphocyte-enriched population was harvested from a 40%-80% Percoll (GE Healthcare) gradient.

### Flow cytometry

Flow cytometry was performed using a standard protocol. Fc receptor blocking was carried out with anti-CD16/32 specific antibodies. A lineage cocktail of antibodies specific for CD3, CD45R, CD19, CD11b, TER-119, Gr-1, CD5 and FcϵRI was used for cLP ILC analyses. Live/Dead Fixable Blue Cell Stain Kit (Invitrogen) stain was used to exclude dead cells from the analysis. The cLP ILC gating strategy is outlined in our recent publications (44, 45). For a complete list of the antibodies used see **Table 1**. A FoxP3 staining kit (ebioscience) was used for intracellular staining of cytokines and transcription factors. In case of cytokine expression analyses, cells were pre-stimulated with 100 ng/ml PMA and 2 µM ionomycin in the presence of 6 µM monensin for 3-4 hours prior to flow cytometry analysis. Samples were acquired using an LSRFortessa™ cell analyser (Becton Dickinson, USA), and all the data were analyzed using FlowJo software (Tree Star, USA).

**Table 1:** Antibody clones and distributors.

Antibody	Clone	Company
α4β7	DATK32	eBioscience
CD25	PC61.5	eBioscience
CD3	17A2	eBioscience
CD5	53-7.3	eBioscience
CD19	1D3	eBioscience
B220	RA3-6B2	eBioscience
CD11b	M1/70	eBioscience/Biolegend
Gr-1	RB6-8C5	eBioscience
Flt3	A2F10	eBioscience
Ter119	TER-119	eBioscience
FcϵRI	MAR-1	eBioscience
CD127	A7R34	eBioscience
NKp46	29A1.4	eBioscience
IL-13	eBio13A	eBioscience
IFNγ	XMG1.2	eBioscience
CD45	30-F11	Invitrogen
CD90.2	5a-8 30-H12	eBioscience BD
IL-5	TRFK5	BD
IL-17A	eBio17B7	eBioscience
KLRG1	2F1	eBioscience
CCR6	29-2L17	eBioscience
NKp46	29A1.4	eBioscience
RORγt	AFKJS-9	eBioscience
NK1.1	PK136	Biolegend

### ILC2 generation in OP9-DL1 system

ILC2p were seeded on OP9-DL1 to generate ILC2 using an established method (52). Briefly, 7,500 cells were co-cultured with mitomycin pre-treated OP9-DL1 in presence of rmIL-7, rmSCF and rmIL-33 (all 20 ng/ml) for 6 days prior to FACS analysis.

### cLP ILC sorting and *in vitro* culture

Single-cell suspensions from colonic lamina propria were stained with fluorescently labelled antibodies and isolated using a BD FACSAria III cell sorter (BD Biosciences). Live CD45^+^ Lin^-^ CD127^+^ ILC FACS sorted as KLRG1^+^ and KLRG1^-^ were cultured in DMEM supplemented with 10% FCS, 1xGlutaMax (Gibco), 50 U/ml penicillin, 50 µg/ml streptomycin, 10 mM HEPES, 1x non-essential amino acids (Gibco), 1 mM sodium pyruvate and 50 μM β-mercaptoethanol (Gibco). 20,000 cells were plated per well of a 96-well plate pre-seeded with OP9-DL1 using an established method (52, 81). The medium was further supplemented with rmIL-7 and rhIL-2 (both at 10 µg/ml) and further recombinant mouse cytokines or anti-CD28 antibody (2µg/ml; clone 37.51) as indicated (all cytokines were used at a final concentration of 10 µg/ml unless indicated otherwise). Cells were harvested and analyzed by flow cytometry after 2 days in culture. FACS sort-derived cells from these conditions were harvested and analyzed without additional pre-stimulation.

### *In vivo* models

DSS-elicted colitis was induced by adding 3 or 5% DSS (36-50 KDa, MP Biomedicals, Ontario, USA) to the drinking water for 5 days and mice were sacrificed 10 days after the beginning of the treatment. To establish a non-colitic control condition, mice were administered sterile drinking water. Regarding all *in vivo* models, body weights and clinical abnormalities were monitored on a daily basis.

For dysbiosis-induced colitis models TRUC mice cecal feces were administered to colitis-free *Rag2* ^-/-^ mice *via* the oral route using a published method and sacrificed 21 days after FMT (45). Regarding all *in vivo* models, body weights and clinical abnormalities were monitored on a daily basis.

### Single-cell RNA-seq analysis

Raw expression matrices were obtained from GEO accession GSE117567 (49) and raw sequencing data were obtained from ArrayExpress accessions E-MTAB-9795 (47) and E-MTAB-11238, (55). Raw reads were mapped to mm10 using CellRanger 6.0.1. UMAP co-ordinates and clustering metadata was obtained from correspondence with the authors of (47, 55), therefore downstream processing steps can be considered identical to those carried out by the respective authors. For the matrices obtained from 49, cells with over 10% reads mapping to mitochondrial genes and cells with less than 400 genes detected were removed. Each matrix was then normalized using SCTransform (82), followed by RunPCA (PCs = 30) and RunUMAP (dims = 30). Shared nearest neighbor and clustering were carried out using FindNeighbours (dims = 30) and FindClusters respectively. NormalizeData was then ran, and this assay was used for downstream visualization and differential expression analysis using the MAST algorithm (83). Pseudotime/trajectory analyses were carried out using monocle3 (84, 85).

### Statistics

Results are expressed as mean ± SEM. Data were analyzed using Student’s t-test using GraphPad Prism 5.0 (GraphPad Inc., USA). ns: non-significant; *p < 0.05; **p< 0.01; ***p<0.001; ****p<0.0001.

### Study approval

All animal experiments were performed in accredited facilities in accordance with the UK Animals (Scientific Procedures) Act 1986 (Home Office Licence Numbers PPL: 70/6792, 70/8127 and 70/7869).

## Data availability statement

The datasets presented in this study can be found in online repositories. The names of the repository/repositories and accession number(s) can be found below: https://www.ebi.ac.uk/arrayexpress/, E-MTAB-9795. https://www.ebi.ac.uk/arrayexpress/, E-MTAB-11213. https://www.ebi.ac.uk/arrayexpress/, E-MTAB-11212.

## Ethics statement

All animal experiments were performed in accredited facilities in accordance with the UK Animals (Scientific Procedures) Act 1986 (Home Office Licence Numbers PPL: 70/6792, 70/8127 and 70/7869). Written informed consent was obtained from the owners for the participation of their animals in this study.

## Author contributions

Study concept and design: J-HS, GL, JN, and NP. Acquisition of data: J-HS, JL, and TZ. Bioinformatics analysis: GB. Data analysis and interpretation: J-HS, GL, RJ, JN, and NP. Obtained funding: GL, NP, and JN. Drafting of manuscript: J-HS. Editing of manuscript: RJ. Study supervision: GL, RJ.

## Supplementary material

Supplementary Figure 1 Gating strategy for cLP ILC. Murine cLP ILC were isolated from *Rag2*-deficient mice for flow cytometry analysis. **(A)** ILC were gated as live single CD45^+^ Lin^-^ CD127^+^ leukocytes. The lineage cocktail contained CD3, CD5, CD19, B220, CD11b, Gr-1, FcϵR1 and Ter119. **(B)** CD90 expression intensity in cLP ILC was evaluated using an FMO control sample.

Supplementary Figure 2 CD90-negative WT cLP CD127^+^ ILC are a source of IFNγ, IL-13 and IL-17A upon DSS treatment of BALB/c mice. cLP ILC from 3% DSS-treated BALB/c WT and *Tbx21* ^-/-^ mice were isolated and stimulated with PMA and ionomycin (3 hours) prior to flow cytometry analysis. **(A)** Frequencies of CD90^hi^, CD90^low^ and CD90^-^ in total CD127^+^ ILC and **(B)** statistical analyses are outlined. **(C)** IFNγ, IL-13 and IL-17A expression in CD90^hi^, CD90^low^ and CD90^-^ CD127^+^ ILC and **(D)** corresponding statistical analyses are shown. Data shown are representative of 4 biological replicates. *p < 0.05; **p < 0.01; ***p<0.001.

Supplementary Figure 3 CD90-negative WT cLP CD127^+^ ILC are a minor source of IFNγ, IL-13 and IL-17A during DSS colitis in C57BL/6 mice. cLP ILC from 3% DSS-treated C57BL/6 WT and *Tbx21* ^-/-^ mice were isolated and stimulated with PMA and ionomycin (3 hours) prior to flow cytometry analysis. **(A)** Frequencies of CD90^hi^, CD90^low^ and CD90^-^ in total CD127^+^ ILC and **(B)** statistical analyses are outlined. **(C)** IFNγ, IL-13 and IL-17A expression in CD90^hi^, CD90^low^ and CD90^-^ CD127^+^ ILC and **(D)** corresponding statistical analyses are shown. **(E)** CD90 co-expression with IL-17A or IFNγ in IL-13^+^ ILC is demonstrated. Data shown are representative of 4 biological replicates.*p< 0.05; **p< 0.01; ***p<0.001.

Supplementary Figure 4 Intestinal CD90-negative CD127^+^ ILC have a predominant type 2 phenotype. cLP CD127^+^ ILC were isolated from untreated C57BL/6 WT and *Tbx21* ^-/-^ mice and stimulated with PMA and ionomycin (4 hours) prior to flow cytometry analysis. **(A)** Frequencies of CD90^hi^, CD90^low^ and CD90^-^ in CD127^+^ ILC and statistical analyses are outlined. **(B)** IL-13, IL-5, IFNγ and IL-17A expression in CD90^hi^, CD90^low^ and CD90^-^ total CD127^+^ ILC and statistical analyses of **(C)** IFNγ and **(D)** IL-17A, **(E)** IL-13 and **(F)** IL-5 expression flow cytometry analyses in CD90^hi^, CD90^low^ and CD90^-^ CD127^+^ ILC are illustrated. **(G)** Statistical analysis of IL-5^+^ IL-13^-^ CD90^hi^, CD90^low^ and CD90^-^ CD127^+^ ILC. Data shown are representative of 3 biological replicates. *p < 0.05; **p< 0.01; ***p<0.001.

Supplementary Figure 5 Characterization of CD90 expression in inflammatory ILC2. cLP CD127^+^ ILC were isolated from untreated C57BL/6 WT and *Tbx21* ^-/-^ mice and stimulated with PMA and ionomycin (4 hours) prior to flow cytometry analysis. Flow cytometry analyses of IL-13 co-expression with **(A)** IL-17A and **(B)** IFNγ in CD90^hi^, CD90^low^ and CD90^-^ CD127^+^ ILC are outlined. Data shown are representative of 3 biological replicates.

Supplementary Figure 6 CD90 and CD127 co-expression in cLP ILC. cLP leukocytes were isolated from untreated C57BL/6 and DSS-treated C57BL/6, BALB/c and Rag2-deficient BALB/c mice. CD127 and CD90 co-expression in lineage-negative leukocytes are shown.

Supplementary Figure 7 Gating strategy for cLP ILC for *in vitro* assay analysis. KLRG1^+^ or KLRG1^-^ CD127^+^ ILC were isolated and stimulated *in vitro* for 48 hours prior to harvest and flow cytometry analyses of KLRG1^+^ or NKp46^+^ ILC, respectively. ILC from these cultures were gated as live single CD45^+^ Lin^-^ leukocytes. The lineage cocktail contained CD3, CD5, CD19, B220, CD11b, Gr-1, FcϵR1 and Ter119.

Supplementary Figure 8 Transcriptome analyses of CD90 expression in intestinal ILC2. A scRNA-seq data set from a published study (49) was employed to analyze CD90 expression across ILC2 isolated from lungs, skin, fat and bone marrow (BM) and its role on the global transcriptional profile. **(A)** A UMAP plot of *Thy1* expression intensity in ILC2 and a trajectory analysis along the CD90 expression intensity was performed in these ILC2. **(B)** Volcano plots comparing gene expression (log2 fold-change and p_adj_) between CD90^high^ ILC versus CD90^low/negative^ ILC2, as annotated in the published data set. The most differentially expressed genes are labelled.
