# Supplementary figures and images for: CD90 is not constitutively expressed in functional innate lymphoid cells
Source: Front Immunol. 2023 Apr 11;14:1113735. doi: 10.3389/fimmu.2023.1113735 (PMC10126679; doi:10.3389/fimmu.2023.1113735)

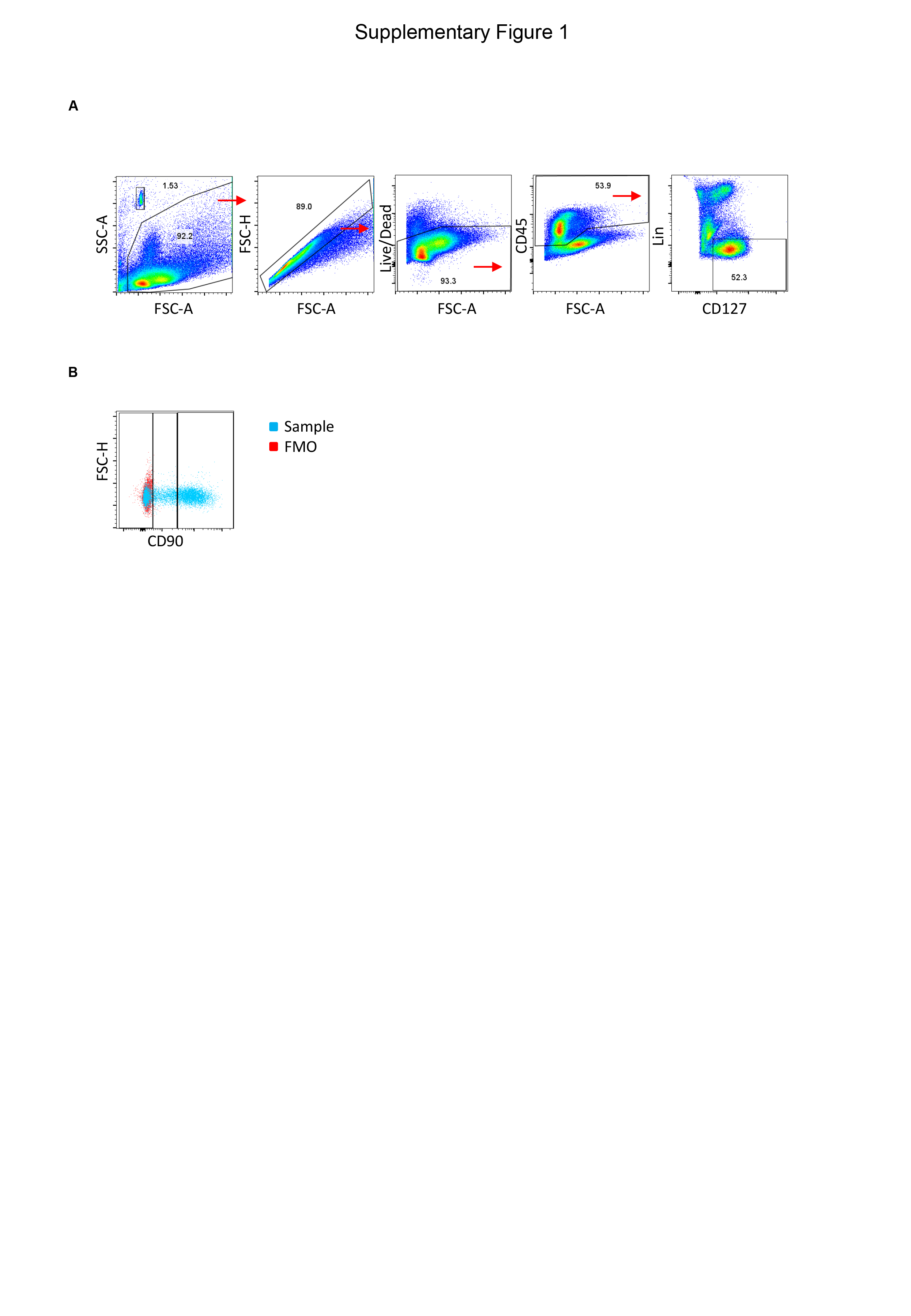

Supplement: Supplementary Figure 1 — Gating strategy for cLP ILC. Murine cLP ILC were isolated from Rag2-deficient mice for flow cytometry analysis. (A) ILC were gated as live single CD45+ Lin- CD127+ leukocytes. The lineage cocktail contained CD3, CD5, CD19, B220, CD11b, Gr-1, FcϵR1 and Ter119. (B) CD90 expression intensity in cLP ILC was evaluated using an FMO control sample. [file Image_1.tif]

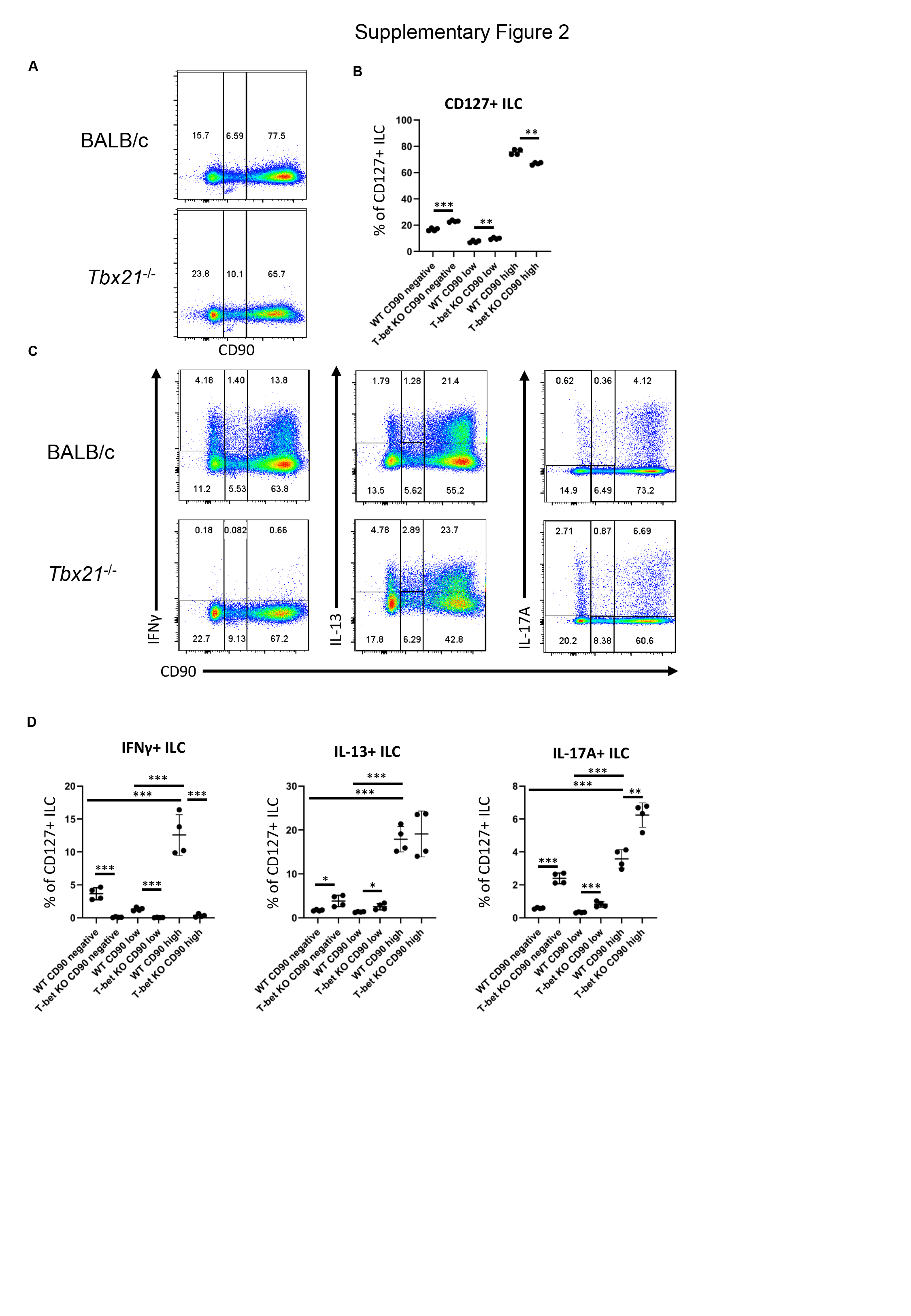

Supplement: Supplementary Figure 2 — CD90-negative WT cLP CD127+ ILC are a source of IFNγ, IL-13 and IL-17A upon DSS treatment of BALB/c mice. cLP ILC from 3% DSS-treated BALB/c WT and Tbx21 -/- mice were isolated and stimulated with PMA and ionomycin (3 hours) prior to flow cytometry analysis. (A) Frequencies of CD90hi, CD90low and CD90- in total CD127+ ILC and (B) statistical analyses are outlined. (C) IFNγ, IL-13 and IL-17A expression in CD90hi, CD90low and CD90- CD127+ ILC and (D) corresponding statistical analyses are shown. Data shown are representative of 4 biological replicates. *p < 0.05; **p < 0.01; ***p<0.001. [file Image_2.tif]

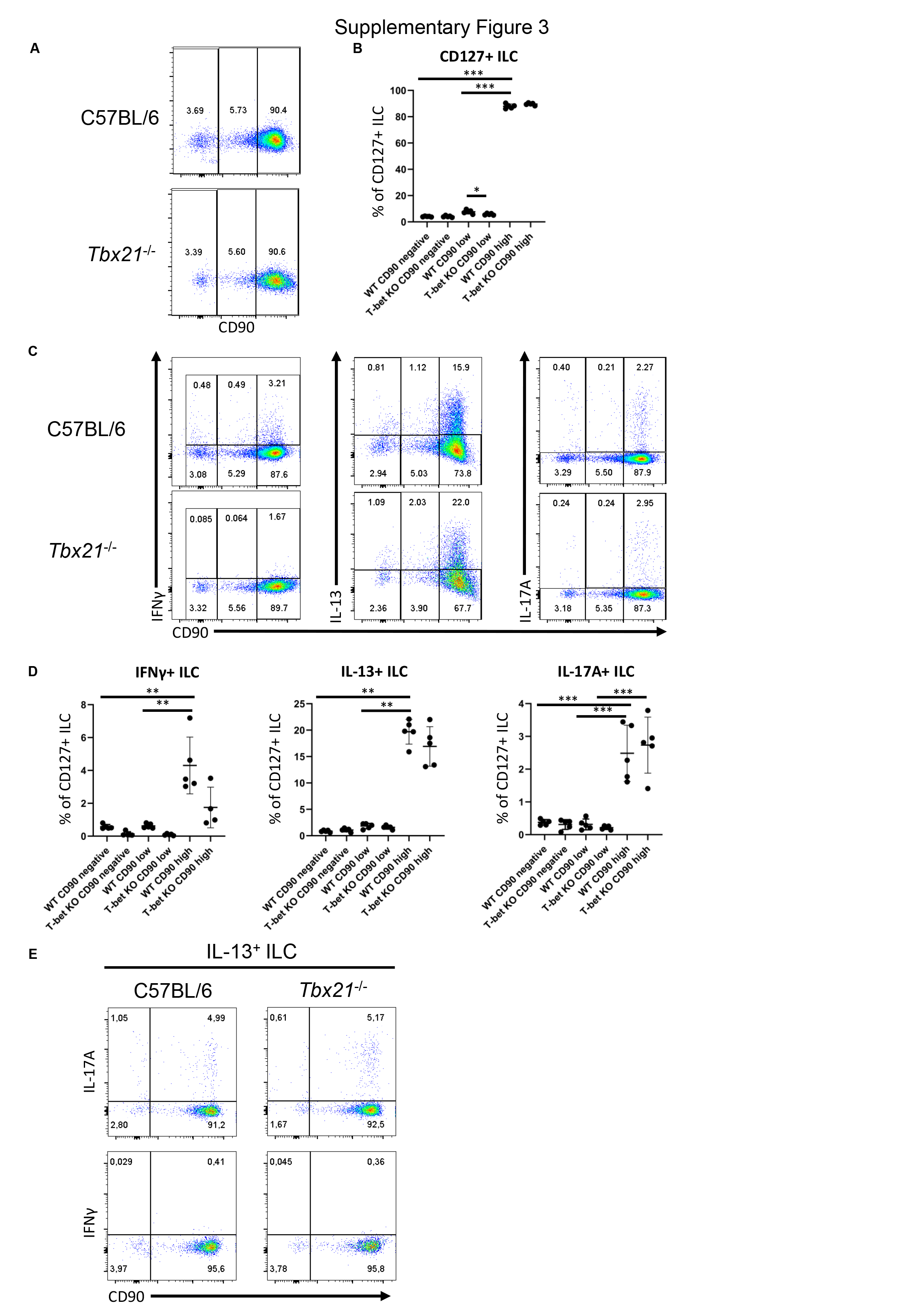

Supplement: Supplementary Figure 3 — CD90-negative WT cLP CD127+ ILC are a minor source of IFNγ, IL-13 and IL-17A during DSS colitis in C57BL/6 mice. cLP ILC from 3% DSS-treated C57BL/6 WT and Tbx21 -/- mice were isolated and stimulated with PMA and ionomycin (3 hours) prior to flow cytometry analysis. (A) Frequencies of CD90hi, CD90low and CD90- in total CD127+ ILC and (B) statistical analyses are outlined. (C) IFNγ, IL-13 and IL-17A expression in CD90hi, CD90low and CD90- CD127+ ILC and (D) corresponding statistical analyses are shown. (E) CD90 co-expression with IL-17A or IFNγ in IL-13+ ILC is demonstrated. Data shown are representative of 4 biological replicates.*p< 0.05; **p< 0.01; ***p<0.001. [file Image_3.tif]

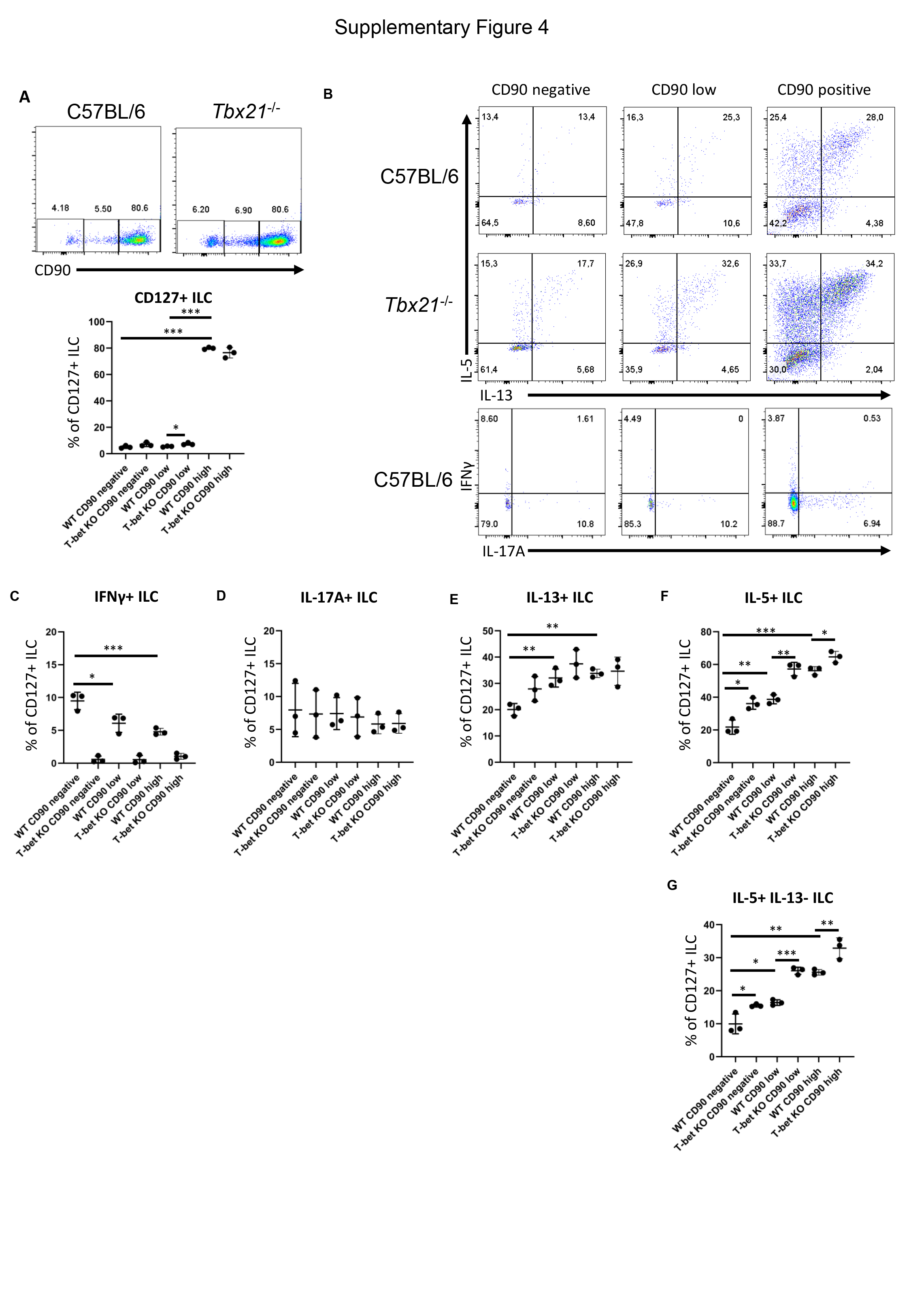

Supplement: Supplementary Figure 4 — Intestinal CD90-negative CD127+ ILC have a predominant type 2 phenotype. cLP CD127+ ILC were isolated from untreated C57BL/6 WT and Tbx21 -/- mice and stimulated with PMA and ionomycin (4 hours) prior to flow cytometry analysis. (A) Frequencies of CD90hi, CD90low and CD90- in CD127+ ILC and statistical analyses are outlined. (B) IL-13, IL-5, IFNγ and IL-17A expression in CD90hi, CD90low and CD90- total CD127+ ILC and statistical analyses of (C) IFNγ and (D) IL-17A, (E) IL-13 and (F) IL-5 expression flow cytometry analyses in CD90hi, CD90low and CD90- CD127+ ILC are illustrated. (G) Statistical analysis of IL-5+ IL-13- CD90hi, CD90low and CD90- CD127+ ILC. Data shown are representative of 3 biological replicates. *p < 0.05; **p< 0.01; ***p<0.001. [file Image_4.tif]

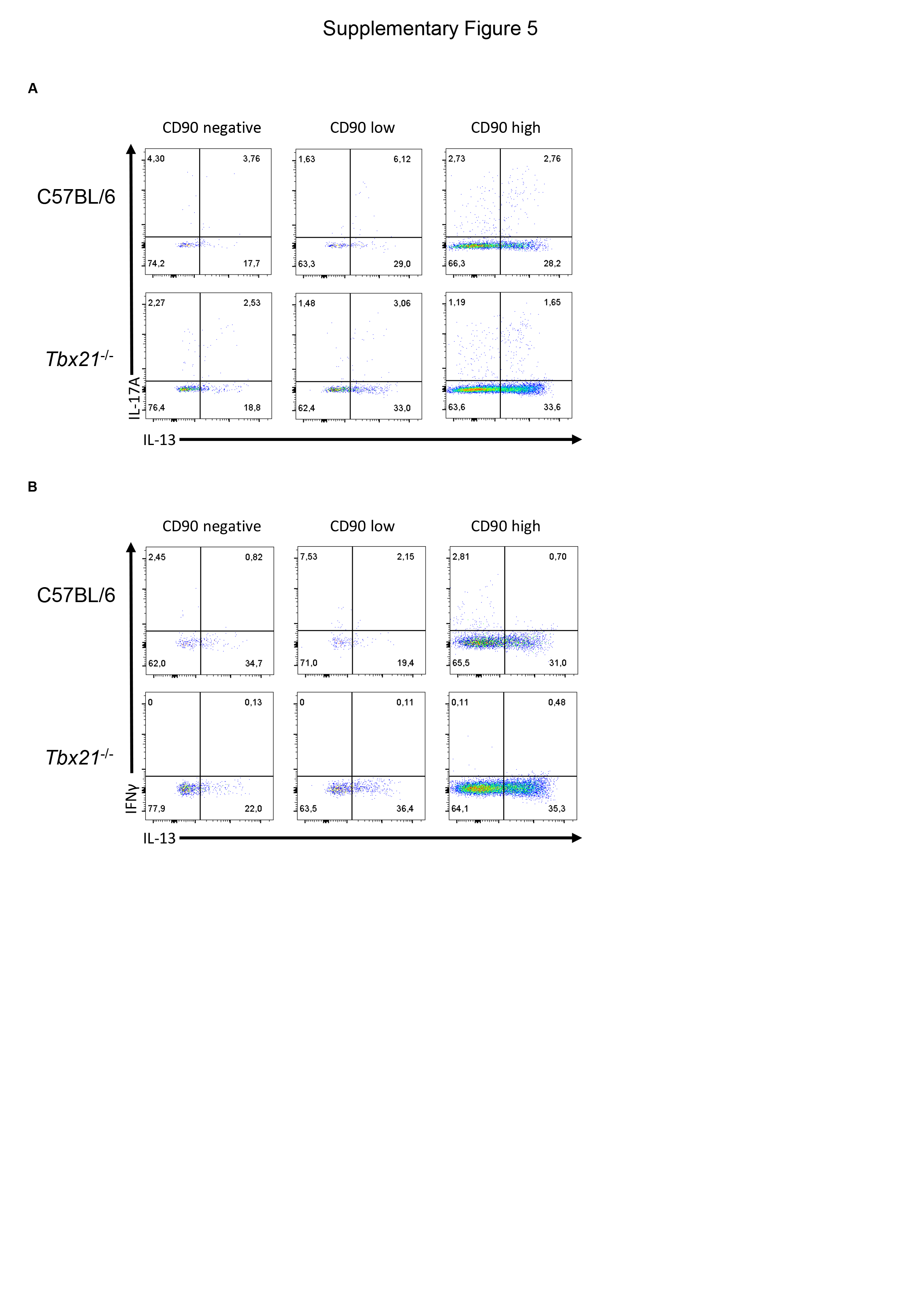

Supplement: Supplementary Figure 5 — Characterization of CD90 expression in inflammatory ILC2. cLP CD127+ ILC were isolated from untreated C57BL/6 WT and Tbx21 -/- mice and stimulated with PMA and ionomycin (4 hours) prior to flow cytometry analysis. Flow cytometry analyses of IL-13 co-expression with (A) IL-17A and (B) IFNγ in CD90hi, CD90low and CD90- CD127+ ILC are outlined. Data shown are representative of 3 biological replicates. [file Image_5.tif]

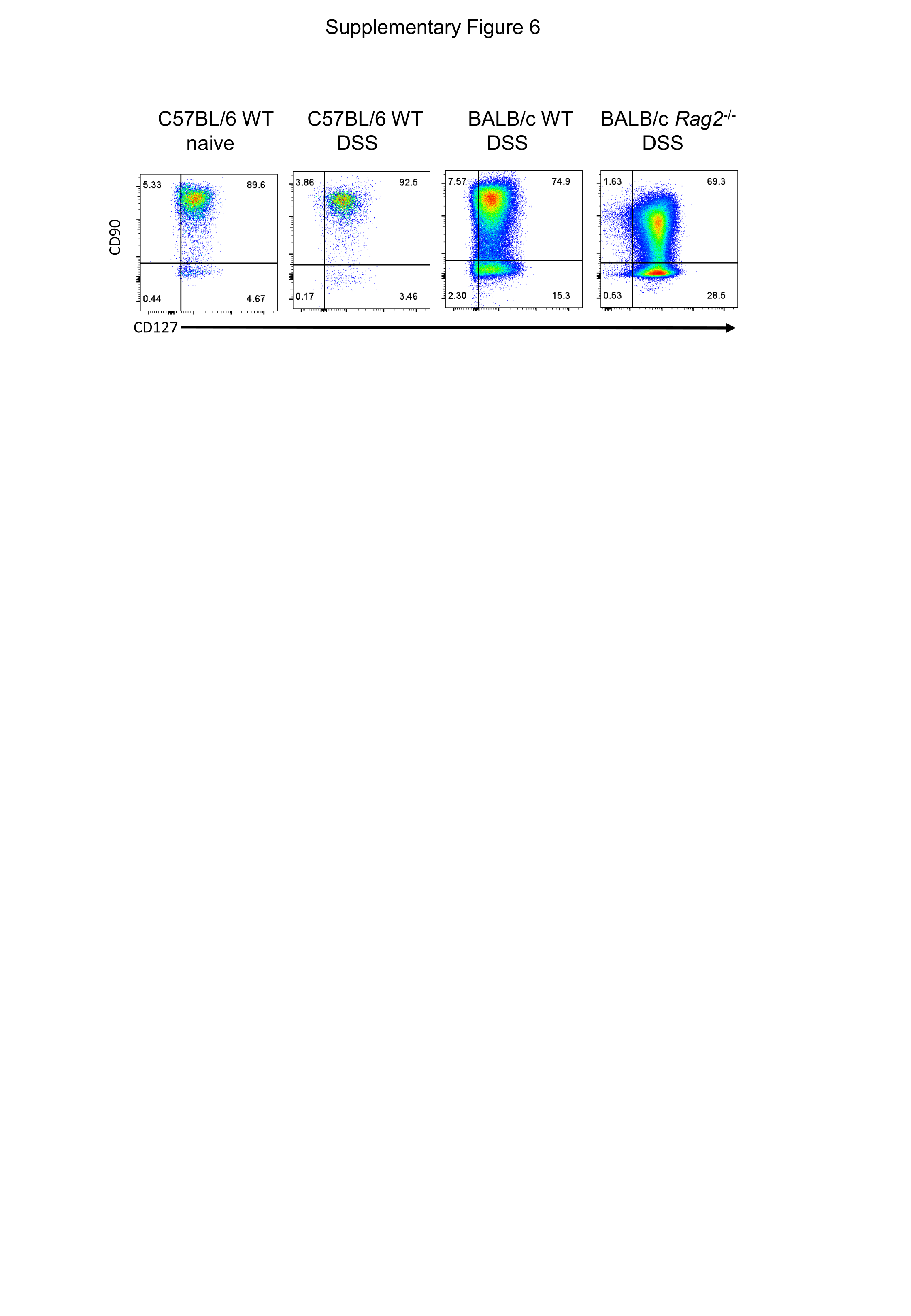

Supplement: Supplementary Figure 6 — CD90 and CD127 co-expression in cLP ILC. cLP leukocytes were isolated from untreated C57BL/6 and DSS-treated C57BL/6, BALB/c and Rag2-deficient BALB/c mice. CD127 and CD90 co-expression in lineage-negative leukocytes are shown. [file Image_6.jpeg]

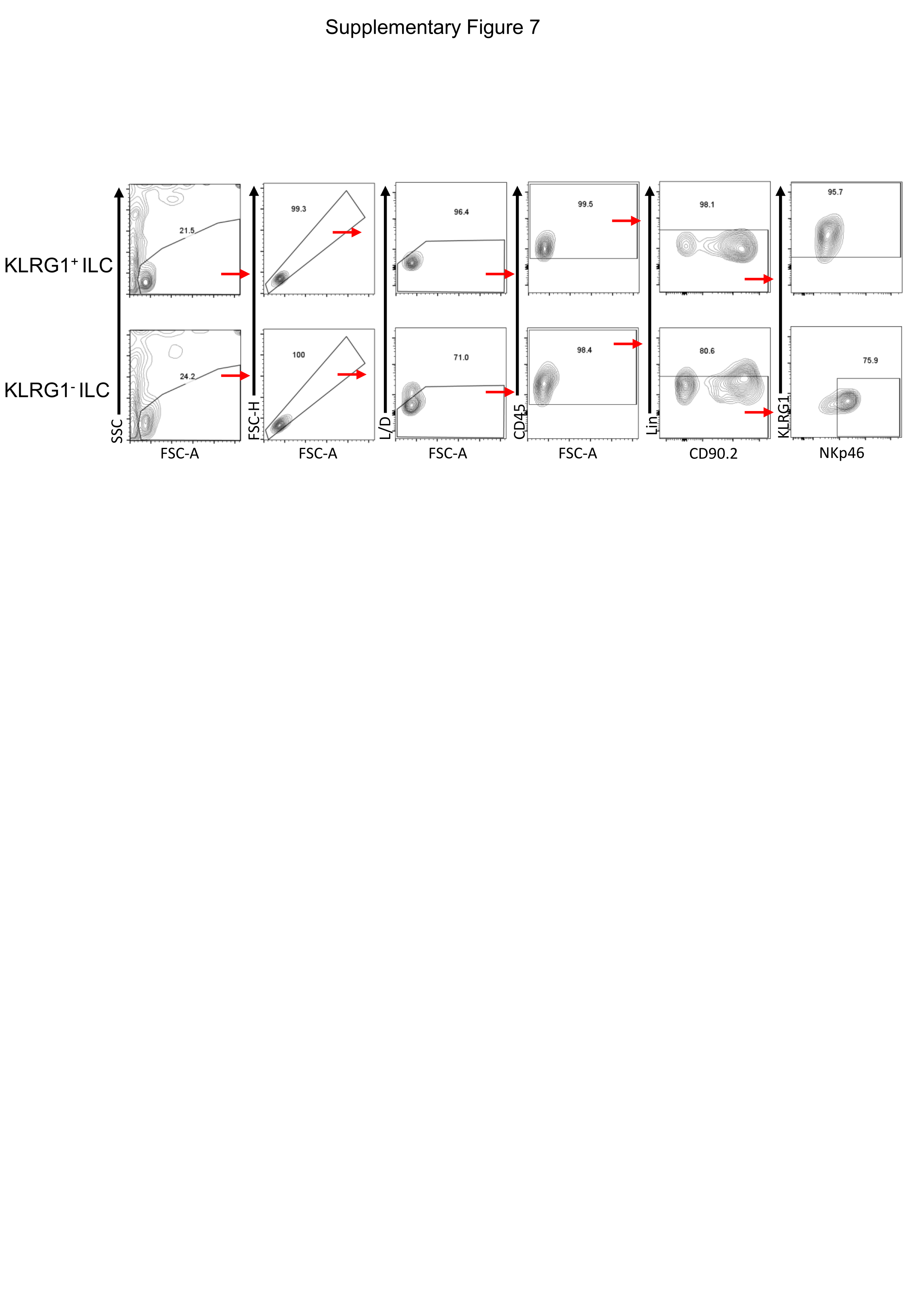

Supplement: Supplementary Figure 7 — Gating strategy for cLP ILC for in vitro assay analysis. KLRG1+ or KLRG1- CD127+ ILC were isolated and stimulated in vitro for 48 hours prior to harvest and flow cytometry analyses of KLRG1+ or NKp46+ ILC, respectively. ILC from these cultures were gated as live single CD45+ Lin- leukocytes. The lineage cocktail contained CD3, CD5, CD19, B220, CD11b, Gr-1, FcϵR1 and Ter119. [file Image_7.jpeg]

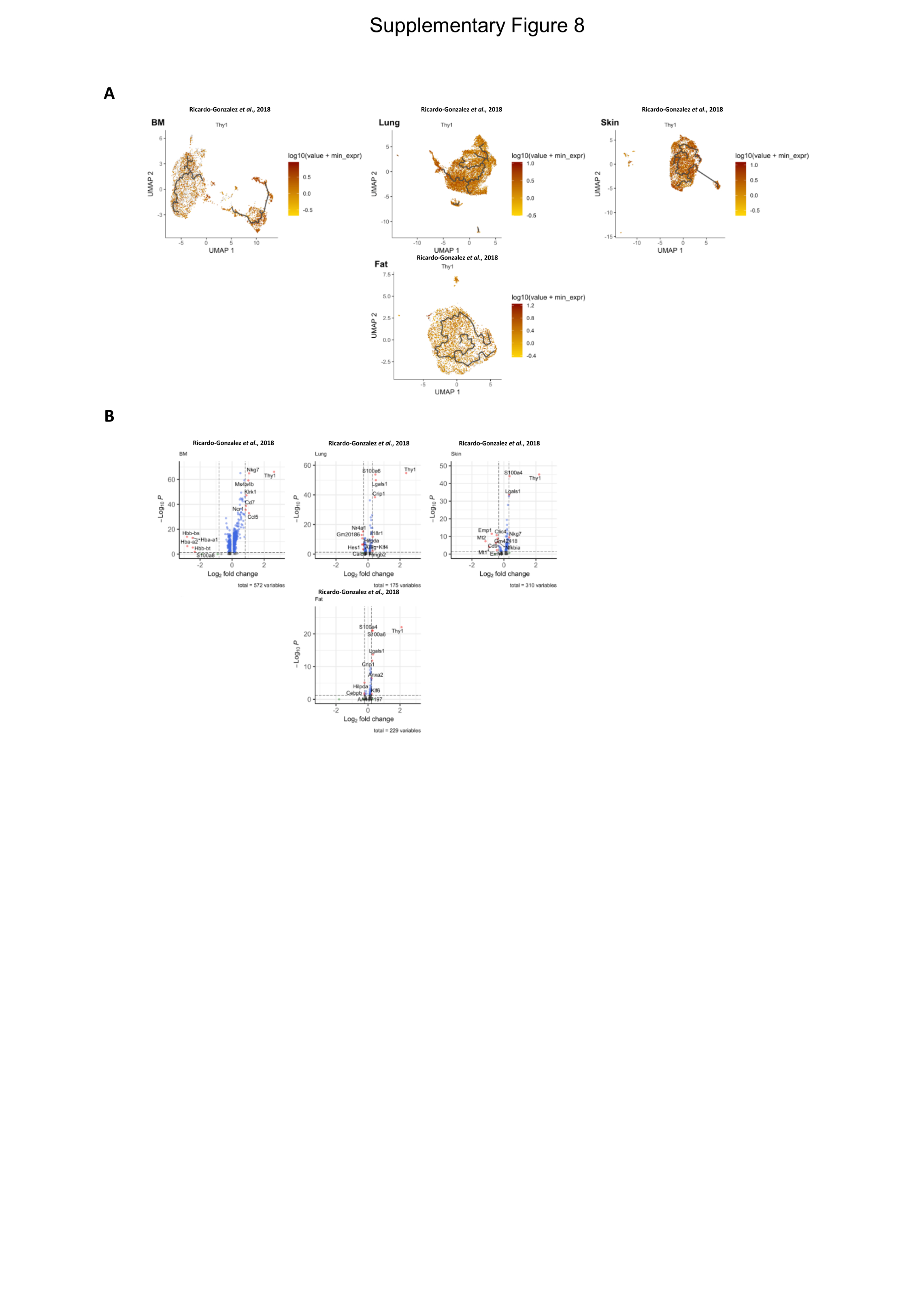

Supplement: Supplementary Figure 8 — Transcriptome analyses of CD90 expression in intestinal ILC2. A scRNA-seq data set from a published study (49) was employed to analyze CD90 expression across ILC2 isolated from lungs, skin, fat and bone marrow (BM) and its role on the global transcriptional profile. (A) A UMAP plot of Thy1 expression intensity in ILC2 and a trajectory analysis along the CD90 expression intensity was performed in these ILC2. (B) Volcano plots comparing gene expression (log2 fold-change and padj) between CD90high ILC versus CD90low/negative ILC2, as annotated in the published data set. The most differentially expressed genes are labelled. [file Image_8.jpeg]
